# A Study on the Health and Wellness of Undergraduate Students (SABES-Grad): methodological aspects of a nationwide multicenter and multilevel study overlapping with the Covid-19 pandemic

**DOI:** 10.47626/2237-6089-2021-0367

**Published:** 2023-03-07

**Authors:** Lauro Miranda Demenech, Lucas Neiva-Silva, Sandra Mara Silva Brignol, Samira Reschetti Marcon, Sônia Maria Lemos, Rafael Miranda Tassitano, Samuel C. Dumith

**Affiliations:** 1 Centro de Estudos sobre Risco e Saúde Universidade Federal do Rio Grande Rio Grande RS Brazil Centro de Estudos sobre Risco e Saúde, Universidade Federal do Rio Grande (FURG), Rio Grande, RS, Brazil.; 2 Programa de Pós-Graduação em Ciências da Saúde FURG Rio Grande RS Brazil Programa de Pós-Graduação em Ciências da Saúde, FURG, Rio Grande, RS, Brazil.; 3 Programa de Pós-Graduação em Psicologia FURG Rio Grande RS Brazil Programa de Pós-Graduação em Psicologia, FURG, Rio Grande, RS, Brazil.; 4 Instituto de Saúde Coletiva Universidade Federal Fluminense Niterói RJ Brazil Instituto de Saúde Coletiva, Universidade Federal Fluminense (UFF), Niterói, RJ, Brazil.; 5 Programa de Pós-Graduação em Enfermagem Universidade Federal de Mato Grosso Cuiabá MT Brazil Programa de Pós-Graduação em Enfermagem, Universidade Federal de Mato Grosso (UFMT), Cuiabá, MT, Brazil.; 6 Escola Superior de Ciências da Saúde Universidade do Estado do Amazonas Manaus AM Brazil Escola Superior de Ciências da Saúde, Universidade do Estado do Amazonas (UEA), Manaus, AM, Brazil.; 7 Departamento de Educação Física Universidade Federal Rural de Pernambuco Recife PE Brazil Departamento de Educação Física, Universidade Federal Rural de Pernambuco (UFRPE), Recife, PE, Brazil.

**Keywords:** Mental health, students, universities, pandemic, Covid-19

## Abstract

**Introduction:**

The academic environment can negatively impact the mental health of undergraduate students, particularly in the context of the coronavirus disease 2019 (Covid-19) pandemic. This study aimed to describe the methodological and operational aspects of a study of the health and well-being of undergraduate students: the Study on the Health and Wellness of Undergraduate Students (SABES-Grad) project.

**Method:**

This was a nationwide cross-sectional study divided across two data collection strategies: a single-center, on-site data collection carried out in 2019 at the Universidade Federal do Rio Grande (FURG) and a multicenter, multilevel, online data collection carried out in 2020/2021 at FURG, the Universidade Federal Fluminense (UFF), the Universidade Federal do Mato Grosso (UFMT), the Universidade do Estado do Amazonas (UEA), and the Universidade Federal Rural de Pernambuco (UFRPE). The main outcomes of interest were depressive symptoms, generalized anxiety, and suicide risk.

**Results:**

A total of 996 students participated in the 2019 data collection (63.8% female; median age of 22 years; response rate of 85.2%) and 5,720 students participated in the 2020/2021 collection (66.7% female; median age of 22 years; response rate of 84.3%). Significant variations in socioeconomic and demographic profiles were observed between the different universities. Approximately one-third of the sample had been tested for Covid-19 in 2020/2021, 7.8% of whom had tested positive.

**Conclusion:**

The SABES-Grad project was the result of collaborative work between several actors from public universities in Brazil. Several aspects of the preparation and execution of this research are discussed in terms of its originality and relevance. Barriers and limitations and strategies adopted to overcome them are also presented.

## Introduction

Higher education institutions have played a historical role in development of science and technology and in qualification of human resources. Nevertheless, the university environment can be challenging and characterized by negative aspects for those who are involved in academic activities. The most common issues include high workload, competition, and stress,^1^ which can all impair the physical and mental health of faculty members and students, particularly undergraduate students.^2 - 4^

Worldwide, one in every five university students is estimated to have a mental disorder of some type.^2^ Psychological distress was shown to be more frequent in this subgroup of the population than among their non-university peers of the same age and as compared to the general population.^4^ A recent meta-analysis reported high prevalence rates of anxiety (37.75%), depression (28.51%), and suicidal behavior (9.1%) among Brazilian undergraduate students.^3^ However, one major shortcoming that impacts extrapolation of data and thus hampers decision-making in higher education institutions is that most studies addressing this topic in Brazil have small sample sizes and focus on health care students, particularly those conducted in the fields of nursing and medicine.^3^

University students have been extensively recruited for epidemiological surveys of mental health because they are easily accessible.^3 , 5^ However, research projects rarely deal with the realities and needs of this population.^6^ Compounding this is an ongoing process of transition from specific admission processes conducted by individual universities (admission exams) to the National Secondary Education Examination (ENEM - Exame Nacional do Ensino Médio), which constitutes an important step towards democratization of access to higher education.^7^ While this strategy has expanded the proportions of freshmen from rather distinct social, economic, and demographic backgrounds, it also poses new challenges to be studied and understood in greater depth.

The current scenario may have been aggravated by what is now recognized as the greatest global health crisis in the modern world: the 2019 coronavirus pandemic (Covid-19). Several non-pharmacological measures were adopted as a strategy to slow the rapid spread of the disease and social distancing was the measure that caused the greatest changes to Brazilians’ daily routine.^8^

Universities across the globe have suspended their on-site activities and migrated to remote teaching.^9^ Such an abrupt change, combined with the extremely adverse outcomes produced by the pandemic (loss of loved ones; political, occupational, and economic instability; and isolation), may together have contributed to increased psychological suffering.^9 - 11^ Importantly, these events may be unevenly perceived among the subgroup of students with greater social vulnerability. Some preliminary studies have indicated high prevalence rates of depressive symptoms, anxiety, and stress among undergraduates during the pandemic period.^12 - 14^ However, an in-depth analysis of the social, economic, and demographic diversity of Brazil concerning this topic has yet to be conducted.

Most studies surveying the mental health of undergraduate students in Brazil have small sample sizes, with regional groups, and some were poorly connected to the daily lives of undergraduates.^3 , 5 , 6^ There is a need for inter-institutional cooperation and for efforts to accurately address the needs of the target population and thus provide information of relevance to researchers, managers of higher education institutions, and the participating undergraduate students themselves.

This study describes the methodological and operational aspects of a nationwide study of the health and well-being of undergraduate students (the Study on the Health and Wellness of Undergraduate Students [SABES-Grad] project). The purpose of the study was to conduct an epidemiological and social diagnosis of the mental health of undergraduate students from public universities in all five administrative regions of Brazil.

## Methods

### Research consortium

The SABES-Grad is an inter-institutional project involving five public universities each located in a different one of Brazil’s five administrative regions: the Universidade Federal do Rio Grande (FURG), the Universidade Federal Fluminense (UFF), the Universidade Federal do Mato Grosso (UFMT), the Universidade do Estado do Amazonas (UEA), and the Universidade Federal Rural de Pernambuco (UFRPE).

The study was conceived in early 2018 on the basis of observations made in the student support department at FURG (the proponent institution). Throughout the second semester of 2018 and the first semester of 2019, research networking and collaboration activities were established between the institutions and researchers. A research project was prepared during this period and submitted to the each institution’s research ethics committee, following the regulations for multicenter studies.

The working group was composed of all actors from a public university in Brazil, specifically: undergraduate students, graduate students (M.Sc. and Ph.D.), newly graduated M.Sc. and Ph.D. students, administrative technicians, Ph.D. professors, and voluntary psychology personnel. Moreover, the multi-professional team comprised specialists in the areas of psychology, nursing, medicine, physical education, nutrition, anthropology, social services, epidemiology, public health, and statistics. The first author was responsible for national coordination of the study and the co-authors were the local coordinators at the participating universities.

This study was originally planned to take place on a face-to-face basis between the second semester of 2019 at FURG (proponent institution) and the first semester of 2020 at UFF, UFMT, UEA, and UFRPE (participating institutions). On-site training sessions were performed by the national study coordination team at all institutions between February and March 2020, except at the UFRPE (where participation started in March 2020). However, face-to-face data collection was only implemented at one of the participating universities (FURG) because of the social distancing measures and suspension of on-site activities caused by the Covid-19 pandemic. As a result, in 2020 the research project had to be adapted to an online format and was submitted for ethical appraisal once again. After approval, the project was conducted at all five institutions. Hence, the SABES-Grad project has a database that allows for a single-center temporal analysis of the higher education context in the pre-pandemic and pandemic periods in Brazil as well as for a cross-sectional multicenter multilevel analysis during the health crisis.

### Study design

The study was divided into two data collection periods, as follows: 1) a cross-sectional, single-center, on-site phase during 2019; and 2) a cross-sectional, multicenter, multilevel, online phase during 2020 and 2021. A panel study was carried out with the data obtained at FURG before and during the pandemic. The results obtained in the 2020/2021 phase made it possible to establish relationships between social, economic, demographic, and contextual diversities as well as to trace the impact of the pandemic on the mental health of undergraduate students in the different regions of Brazilian.

### Study sites

Participating institutions were selected through convenience sampling based on contacts with researchers in the field. Thus, the present study does not envision including a representative sample of Brazil but rather prioritizes the social, economic, demographic, and cultural diversity existing in the country. Table 1 shows a list of the selected institutions, their characteristics and locations, and the data collection periods.

**Table 1:** Description of study centers – The SABES-Grad project, Brazil, 2021

Institution (approximate number of undergraduates)	City/state (approximate number of inhabitants)	Region	Data collection period
FURG (9,000)	Rio Grande/RS (200 thousand)	South	September 2019-November 2019* October 2020-January 2021
			
UFF (23,000)	Niterói/Rio de Janeiro (400 thousand)	Southeast	November 2020-January 2021
			
UFMT (32,000)	Cuiabá/Mato Grosso (600 thousand)	Mid-West	September 2020-November 2020
			
UFRPE (15,000)	Recife/Pernambuco (1.6 million)	Northeast	February 2021-May 2021
			
UEA (20,000)	Manaus/Amazonas (2 million)	North	November 2020-March 2021

FURG = Universidade Federal do Rio Grande; SABES-Grad = Study on the Health and Wellness of Undergraduate Students; UEA = Universidade do Estado do Amazonas; UFF = Universidade Federal Fluminense; UFMT = Universidade Federal do Mato Grosso; UFRPE = Universidade Federal Rural de Pernambuco.

* FURG was the only university that collected data face-to-face in 2019, before on-site activities were suspended because of the coronavirus disease (Covid-19) pandemic.

### Target population

The target population of this study consisted of undergraduate students enrolled at any of the participating institutions. Students over the age of 18 years at data collection and properly enrolled at the participating institutions were considered eligible. Those who had dropped out or interrupted their undergraduate course or had physical and/or cognitive limitations that prevented them from understanding and filling out the self-administered questionnaire were considered ineligible.

### Sample size calculation

Two sample size calculations were performed using the Epi Info 7 program, for data collected on-site (2019) and online (2020/2021).

First, the minimum number of respondents required to accurately estimate the prevalence of the outcomes at each institution was determined. Table 2 lists the statistical parameters used for the sample size calculation. An additional 50% was included in the sample to allow for the design effect ( *deff* ), which was estimated at 1.5 (intraclass correlation coefficient = 0.02; conglomerate mean size = 20^15^). Thus, the following numbers of participants were needed: 770 from FURG; 798 from UFF; 803 from UFMT; 788 from UFRPE; and 795 from UEA.

**Table 2:** Descriptive sample size calculations (2019 and 2020/2021 data collection) - the SABES-Grad project, Brazil, 2021

	Depression	Anxiety	Suicide risk	Total
Expected (%)	25	30	15	-
Margin of error (p.p.)	4	4	3	-
				
University				
FURG (n = 9,000)	644	716	770	770
UFF (n = 23,000)	663	740	798	798
UFMT (n = 32,000)	666	744	803	803
UFRPE (n = 15,000)	656	732	788	788
UEA (n = 20,000)	660	738	795	795

Sample size calculations were conducted taking into account a design effect ( *deff* ) of 1.5.

% = expected prevalence of each outcome; FURG = Universidade Federal do Rio Grande; n = approximate number of undergraduate students enrolled at the institution. p.p. = percentage points; SABES-Grad = Study on the Health and Wellness of Undergraduate Students; UEA = Universidade do Estado do Amazonas; UFF = Universidade Federal Fluminense; UFMT = Universidade Federal do Mato Grosso; UFRPE = Universidade Federal Rural de Pernambuco.

A second calculation was performed to estimate the sample size required to provide statistical power for significant associations in isolated samples (from each participating institution). The outcome with the lowest expected prevalence (suicide risk at 15%) and estimates for some of the main individual exposures (social support, food insecurity, discrimination, illicit drug use, stress, and per capita family income) were used in the calculation. A total of 1,089 participants were needed from each institution ( Table 3 ). Therefore, this was the sample size used for all institutions, because it was larger than the size estimated by the calculation for prevalence rates.

**Table 3:** Sample size calculations for associated factors using the outcome with the lowest expected prevalence (suicide risk = 15%) – the SABES-Grad project, Brazil, 2021

Exposure	Proportion of exposed (%)	Ratio exposed: Unexposed	Prevalence of outcome in unexposed (%)	Prevalence ratio	Subtotal	Total*
Low social support	25	1:3	10	2.0	551	965
Food insecurity	33	1:2	10	2.0	567	992
Discrimination	30	1:2	12	2.0	593	1,038
Last month illicit drug use	20	1:4	12	2.0	617	1,080
High levels of stress (highest quartile)	25	1:3	9	2.0	622	1,089
Low per capita family income (lowest quartile)	25	1:3	9	2.0	622	1,089

SABES-Grad = Study on the Health and Wellness of Undergraduate Students.

* The subtotals were increased by 10% to account for possible losses and refusals, by 15% to control for confounding factors, and by 50% to account for the design effect (1.5).

### Study variables and research instruments

#### Individual outcomes

Undergraduate students were asked about anxiety symptoms,^16^ depressive symptoms,^17 , 18^ suicide risk,^19^ use of alcohol, tobacco, and illicit drugs,^20^ and stress levels,^21^ to enable assessment of their mental health with instruments validated for use in Brazil. We note that social anxiety symptoms,^22^ and quality of life^23^ were only assessed in the 2019 dataset. These instruments were removed from the 2020/2021 data collection to reduce the length of the final instrument, because it was necessary to include additional questions about the Covid-19 pandemic.

Table S1, available as online-only supplementary material, presents the outcomes, the instruments used, and the operationalization of variables. It is intended to set up a repository with some of the documents produced by the study, including the questionnaire, which will be freely accessible to researchers wishing to investigate the topics covered herein.

#### Individual exposure

An important differentiating feature of the SABES-Grad project is that it produced a broad and detailed description of social, economic, demographic, and academic aspects of undergraduate students and thus produced a report presenting the interrelationship between mental health/disorders and their social determinants. The exposures and data collection methods and their respective operationalizations can be seen in detail in Table S2, available as online-only supplementary material.

The first section contained questions about biological sex, gender identity, age, weight, height, relationship status, self-report skin color, religion, income (individual and family), and parents’ education level. In the 2019 data collection, physical activity and sedentary behavior were measured using the International Physical Activity Questionnaire (IPAQ), which has been translated and validated for use in Brazil.^24^ In the 2020/2021 data collection, only physical activity was assessed (due to the need to reduce the length of the final instrument), using the questions “Considering the last 7 days, on how many days did you do physical activity?” and “On the days you did physical activity, how long did it last on average (in minutes)?” Questions addressing the individual’s socioeconomic position and adverse childhood experiences were also included^25^ (in 2019 only, for the same reason). In addition, the participant’s current housing situation was also surveyed, covering academic migration, with whom they were living, type and condition of housing, fear of violence in the neighborhood where they lived, and adequate access to essential services. Participants were also asked about their perceptions of access to, and use of, medical and psychological services.

The second section contained questions about academic aspects, more specifically about the university at which the student was enrolled (FURG, UFF, UFMT, UFRPE, or UEA); current undergraduate course; shift; year of admission; duration of the course; whether the current course was their first (or if they had already started/finished another course); year/semester at time of data collection; the number of modules they had failed in the previous semester; whether their current course was what they had wanted to study at the time of admission; and how satisfied they were with their undergraduate course.

The third section addressed sexual behavior characteristics and history of diagnosis of sexually transmitted infections (STIs). The questions were prepared based on the Survey of Knowledge, Attitudes, and Practices in the Brazilian Population (PCAP- Pesquisa de Conhecimento, Atitudes e Práticas na População Brasileira) conducted by the Brazilian Ministry of Health.^26^

The fourth block surveyed experiences with discrimination (EDS - Everyday Discrimination Scale^27^), food insecurity (abridged version of the Brazilian Food Insecurity Scale [EBIA - Escala Brasileira de Insegurança Alimentar^28^]), social support (SSS - Social Support Scale^29 , 30^), and sleep quality (MSQ- Mini Sleep Questionnaire^31^). All instruments employed in our study are validated for use in Brazil.

#### Individual exposure related to the pandemic

In the 2020/2021 data collection, an additional section was included containing questions related to the Covid-19 pandemic (Table S2). These questions addressed some possible impacts of the pandemic on work/occupation and family income, compliance with recommendations for social distancing, changes to the routine of activities, inflow and outflow of people in the house, number of days that they had left their house in the previous 15 days, change in the duration and quality of sleep due to the pandemic, and access to information about Covid-19 (number of days in the previous week they had searched for information, how many times a day and, on average, how many minutes they had spent searching for information each time).

Fear of Covid-19 was assessed using the Fear of Covid-19 Scale (FCV-19S),^32^ which has been previously translated and validated for use in Brazil.^33 , 34^ Questions were also asked about taking diagnostic tests for Covid-19, test results (negative or positive), outcomes related to positive testing (staying at home, care via teleservices, visit by a health professional, hospitalization, admission to an intensive care unit [ICU]), presence of risk factors for complications associated with Covid-19, and illness and death of loved ones caused by the disease.

#### Contextual exposure

The SABES-Grad project also aimed to assess the influence of social aspects on production of psychological suffering. Importantly, individual outcomes occur within the subject’s micro and macro social, economic, demographic, and cultural context.^35 , 36^ Context-level information was incorporated to address these nuances, resulting in a multi-level database with multiple exposure layers, as follows: individual variables (discussed in the previous section); university-related variables (number of mental health professionals – gross and proportional to the number of students; a resource applied to student care); variables related to the municipalities/states in which participants were residing at the time of data collection (economic inequality through the Gini coefficient^37^; Human Development Index [HDI]^38^; Brazilian Deprivation Index [IBP - Índice Brasileiro de Privação]^39^; impact of the Covid-19 pandemic based on the incidence of infection and mortality rates obtained from the Coronavirus Panel^40^; and the impact of the pandemic on unemployment rates and regional variations in income obtained from the Instituto Brasileiro de Geografia e Estatística [IBGE] website^41^).

Other exposure layers were defined based on secondary data. Hence, while the contextual exposures included in the database have already been defined, future possibilities can be tested – as long as they deal with exposures that occurred at the same time as the individual data collection.

## Logistics and procedures

### Data collection in 2019

The sampling process was carried out systematically by clusters in a single stage based on the list of all classes contained in the university system. In our study, a class was defined as a group of students enrolled on the same module. Based on previous reports, student classes have approximately 20 undergraduates.^15 , 42^ Thus, 55 classes (1,089 ÷ 20) would need to be surveyed to obtain the required sample size. Considering the possibility of having individuals enrolled in two or more classes and/or aged under 18, five more classes (10%) were added. Therefore, 60 classes were systematically drawn from the university system, according to a previously calculated selection interval.

The 2019 fieldwork was conducted during September and November at FURG only. The professors in charge of the classes that had been selected were contacted to schedule visits to collect data from students regularly enrolled on the module. Class visits were standardized, starting with presentation of the research objectives and confidentiality measures. Those who agreed to participate signed an Informed Consent Form, after which the questionnaire was administered. On completion of administration, participants deposited their completed questionnaires into a sealed urn, to increase confidentiality and reliability of answers. Each class was visited at least twice. After these two attempts, classes with more than 10 losses were visited once again. Individuals were considered as losses if they were not found in any of the visits or they refused to participate. Data were double-entered by different professionals into the Epidata 3.1 program.

### Data collection in 2020/2021

Due to social isolation, distancing recommendations, and cancellation of on-site activities during the Covid-19 pandemic, on-site data collection could not be continued since university activities were suspended and resumed remotely. Thus, after due adjustments and approval by the ethics committees, online data collection was implemented. The questionnaire was imported to the Research Electronic Data Capture (REDCap) platform and made available to all undergraduate students at the participating universities.

The work at the five universities took place between September 2020 and May 2021. First, an invitation with the questionnaire link was sent through messages via the universities’ systems or the e-mails of course coordinators, module professors, and academic directories, or directly to the students enrolled by the institutions’ information systems, with formal consent of the university management. Second, extensive publicity was sent via social media accounts belonging to the institutions and research groups involved in the study, as well as through direct messages via instant messaging applications. If the first part of the form was completed, the questionnaire was considered valid (questions about the link with the university and current undergraduate course). The questionnaire was made available for 2 months at each institution and could be kept available for another 2 months if the number of participants indicated in the sample calculation had not yet been reached ( Table 1 ).

## Ethical considerations

The research project was submitted to the ethics committees at the participating institutions in accordance with National Health Council Resolutions 346/2005 (which deals with processing of multicenter projects)^43^ and 466/2012 (which establishes the ethical precepts to be respected in research with human beings).^44^ The 2019 study was approved under protocol no. 3.474.128 (CAAE: 5159119.1.1001.5324) and the 2020/2021 study was approved under protocol numbers 4.146.935 (FURG), 4.351.740 (UFF), 4.229.295 (UFMT), 4.417.328 (UFRPE), and 4.335.298 (UEA) (CAAE: 24520719.3.2003.5016). All participating individuals signed a consent statement.

One of the main risks of our study concerns the possible discomfort generated by the questionnaire, especially the questions about symptoms of anxiety, depression, suicide risk, and adverse experiences (e.g., situations of sexual violence). In the 2019 data collection, all fieldwork personnel were properly trained in mental health support and were fully monitored and supported by psychologists who remained on duty during all three shifts throughout data collection. For the 2020/2021 data collection, a remote psychology service was made available to all participants who expressed interest through the available contacts (e-mail and/or telephone). A team of volunteer psychologists linked to FURG’s Psychology Services Center (Centro de Atendimento Psicológico) was available throughout the fieldwork for participants from all universities. In addition to the mental health services available at the clinic run by the UFF School of Psychology (Faculdade de Psicologia), the local coordination at the UFF also assembled a mental health care team made up of volunteer psychologists and psychiatrists, organized in three shifts to provide support, care, and referral. Participants were also offered a list of various face-to-face and remote healthcare options that they could access directly, free of charge, if they preferred this alternative. Contacts were also provided for the Center for the Appreciation of the Value of Life (CVV - Centro de Valorização da Vida).

## Results

A total of 1,169 undergraduate students were considered eligible among the members of the 60 classes selected for the 2019 study. The responses of 996 students were validated, which corresponds to a response rate of 85.2%. The general statistical power of the 2019 study was 98.6%, considering the same parameters used for the sample calculation of associated factors (exposed/unexposed ratio of 1:3, prevalence ratio of 2.0, and significance level of 0.05).

A total of 5,720 students participated in the 2020/2021 study, 4,822 of whom answered all the questions on the form (response rate: 84.3%). The distribution of students according to their institutions was as follows: FURG – 1,437 participants (1,209 forms completed; 84.1%); UEA – 1,101 participants (933 forms completed; 84.8%); UFF – 1,132 participants (977 forms completed; 86.3%); UFMT – 1,762 participants (1,450 forms completed; 82.3%); and UFRPE – 288 participants (253 forms completed; 87.9%). The overall statistical power of the study using the same parameters was 100%. When observed individually by institution, the study’s statistical power was as follows: FURG – 99.9%; UEA - 99.2%; UFF - 99.3%; UFMT – 99.9%; and UFRPE – 61.7%.

Table 4 lists the characteristics of the study participants. The 2019 FURG sample was composed mostly of female students (63.8%), with white self-report skin color (73.6%). Their median age was 22 years (interquartile range [IQR] 20-26) and their per capita household income was R$1,200 (IQR 700-2,000).

**Table 4:** The SABES-Grad project sample characteristics for 2019 and 2020/2021 data collections, Brazil, 2021

2019		2020/2021						
	
Characteristics	FURG	Characteristics	FURG	UEA	UFF	UFMT	UFRPE	Total
	
	n (%)		n (%)	n (%)	n (%)	n (%)	n (%)	n (%)
Sex (N = 994)		Sex (N = 5,720)						
Male	359 (36.1)	Male	428 (29.8)	405 (36.8)	364 (33.2)	590 (33.5)	115 (39.9)	1,902 (33.3)
Female	635 (63.8)	Female	1,009 (70.2)	696 (63.2)	768 (67.8)	1,172 (66.5)	173 (60.1)	3,818 (66.7)
								
Skin color (N = 994)		Skin color (N = 5,720)						
White	732 (73.6)	White	974 (67.7)	334 (30.3)	645 (56.9)	702 (39.8)	109 (37.9)	2,764 (48.3)
Black	89 (9.0)	Black	182 (12.7)	76 (6.9)	175 (15.5)	293 (16.6)	51 (17.7)	777 (13.6)
Brown, yellow, or other	173 (17.4)	Brown, yellow, or other	281 (19.6)	691 (62.8)	312 (27.6)	767 (43.6)	128 (44.4)	2,179 (38.1)
			
	**Median (IQR)**		**Median (IQR)**	**Median (IQR)**	**Median (IQR)**	**Median (IQR)**	**Median (IQR)**	**Median (IQR)**
			
Age (years) (N =996)	22 (20-26)	Age (years) (N = 5,719)	23 (21-30)	22 (20-25)	23 (21-26)	22 (20-26)	22 (20-26)	22 (20-26)
Per capita family income (Reais) (N = 836)	1.200 (700-2.000)	Per capita family income (Reais) (N = 5,696)	800 (450-1,333)	750 (357-1,666)	1,300 (700-2,500)	1,100 (650-2,000)	644 (400-1,200)	1000 (500-1,800)
	
			**n (%)**	**n (%)**	**n (%)**	**n (%)**	**n (%)**	**n (%)**
			
		Covid-19 test (N = 5,486)						
		Not tested	1,079 (78.6)	621 (58.5)	764 (69.5)	1,284 (76.9)	226 (79.6)	3,974 (72.4)
		Tested (negative result)	240 (17.5)	276 (26.0)	251 (22.8)	271(16.3)	44 (15.5)	1,082 (19.7)
		Tested (positive result)	53 (3.9)	165 (15.5)	84 (7.7)	114 (6.8)	14 (4.9)	430 (7.8)

% = prevalence by subgroup; FURG = Universidade Federal do Rio Grande; IQR = interquartile range (percentiles 25 to 75); n = total number of observations by subgroup; N = total number of observations in the study; SABES-Grad = Study on the Health and Wellness of Undergraduate Students; UEA = Universidade do Estado do Amazonas; UFF = Universidade Federal Fluminense; UFMT = Universidade Federal do Mato Grosso; UFRPE = Universidade Federal Rural de Pernambuco.

The sample for the 2020/2021 multicenter study was also mostly female (66.7%), with variations in sex proportion by institution. The largest proportion of females was observed at FURG (70.2%) and the smallest was at UFRPE (60.1%). In total, 48.3% of the participants self-reported white skin color, while the frequencies of self-reported black and brown/yellow/other skin color were 13.6 and 38.1%, respectively. Nevertheless, there was an important variation in skin color – the highest proportion of white individuals was observed among students at FURG (67.7%) and the lowest at UEA (30.3%). Black skin color was most frequent among students at UFPRE (17.7%) and least frequent at UEA (6.9%). Finally, self-reported brown, yellow, or other skin color was observed most frequently at UEA (62.8%) and least frequently at FURG (19.6%). The median age of all participants was 22 years (IQR 20-26), which was quite homogeneous when observed by institution. The median per capita family income of the total sample was R$1,000 (IQR 500-1,800). The institution with the lowest per capita family income was UFRPE (R$ 644 [IQR 400-1,200]), while the institution with the highest per capita income was UFF (R$ 1,300 [IQR 700-2,500]) ( Table 4 ).

The prevalence of Covid-19 testing for the whole sample was 27.6% (19.8% had negative results and 7.8% had positive results). The institution with the highest proportions of both testing and of positive results was UEA (41.5% – 26.0% negative; 15.5% positive) ( Table 4 ).

## Discussion

Cross-sectional studies are relevant tools for epidemiological diagnoses of current situations. Although there are limitations to consider, especially regarding the impossibility of establishing temporality and causality, cross-sectional designs are low-cost while providing useful information for understanding a problem and guiding decision-making.^45^ Associated with this, if repeated over time, this type of study allows for comparisons of different points in time and for drawing inferences from the data. When a large variability of contexts is included, the generalizability of findings is substantially increased.

The SABES-Grad project had to be interrupted due to the Covid-19 pandemic, given the social distancing measures and the suspension of on-site activities at universities in Brazil and worldwide.^8 , 9^ At first, this was seen as a major problem by our group, which had been designing the study since 2018. Setbacks are very likely to occur in almost every research project. Even though researchers are trained to handle such situations, one could hardly foresee the barriers imposed by the Covid-19 pandemic. The research redesign was only possible due to the prior well-established interinstitutional collaboration and cohesion of the professionals involved. Therefore, this difficult background was also seen as an opportunity to assess differences in the levels of psychological distress between the pre- and ongoing pandemic contexts. This could provide valuable information, even if from only one of the participating institutions. Likewise, the multicenter study allowed us to capture the most varied situations of 5,720 undergraduate students in Brazil from the most diverse cities in the 27 states of the country. Thus, different individual and contextual experiences make up a database that encompasses the great variability of the university population, with great potential for analysis of the impact of the pandemic on the mental health of the academic population and to support the formulation of hypotheses for further research. To the best of our knowledge, this is the biggest and most comprehensive survey of Brazilian undergraduate students’ health and wellness during the Covid-19 pandemic. The results will provide valuable information for key stakeholders to prepare a response to tackle the impacts of the pandemic on this population.

Physical and mental health is largely determined by social, economic, demographic, and cultural aspects in a complex interrelationship between individual characteristics and background influences.^35 , 46 , 47^ The in-depth assessment of social determinants enhances the originality and relevance of the study and may help to shed light on the relationship between social position and the physical and mental health outcomes related to this health crisis. As shown in the results, there are differences in socioeconomic and demographic structures between the study participants (sex, income, and skin color), which highlights the wide diversity of the university population in Brazil. The ability to incorporate regional secondary data into the database, including layers of contextual exposures, will allow for multi-level analysis to explore the influence of social determinants at the individual and contextual level simultaneously, thereby reducing the possibility of individualistic and ecological fallacies.^36^ It also makes it possible to measure the existence of associations between the severity of the pandemic in the place where the person was experiencing it (regional incidence and mortality by Covid-19) and their levels of psychological suffering. This may provide clear evidence of the possible impacts of the pandemic on the mental health of undergraduate students.

This study has important limitations to consider, namely: (i) the interruption of on-site data collection due to sanitary restrictions in the sudden context of the pandemic, which led to suspension of activities in Brazilian universities from March 2020. Hence, comparisons of the data from before and during the pandemic from one of the institutions (FURG) should be made with caution, since important methodological aspects were handled differently due to the conditions imposed, including sampling (probabilistic versus by invitation) and the data collection format (in-person questionnaire vs. online form); (ii) in the 2020/2021 collection, the number of UFRPE participants was significantly lower than the numbers of participants from the other institutions. We reason that this may have occurred for two reasons: 1) UFRPE was the only university that did not receive on-site training and the lack of human contact, which is important to create a bond between the researchers and the institution (both its management and the target population), may have impaired dissemination of the project and hindered invitation through institutional means that could have greater reliability for the person being invited)^48^; and 2) UFRPE was the last institution at which data collection was started because of the timeline of project approval by its institutional research ethics committee; (iii) during the Covid-19 pandemic, several studies were conducted in this format and it was initially well-tolerated by the study subjects. However, over time, potential participants seemed to be unwilling to participate due to the large number of studies being conducted in this format. As the statistical power of this data collection was low (61.7%), isolated analyses must be carried out with caution.

Lastly, experts indicate that during the Covid-19 era, there was also a pandemic of online research,^49 , 50^ which points to new horizons for research in the health sciences as well constituting a warning to exercise caution with regard to methodological rigor and generalization of results. In particular, the possibility of selection bias should be considered, since these studies basically include people with internet access who are willing to participate.^51 , 52^ Strong pre-fieldwork preparation, including technical training of personnel, strengthening of human and professional bonds, and facilitation of institutional relationships may help overcome these issues, and are therefore recommended for future research on this topic.

## Conclusion

The SABES-Grad project is a collaborative effort involving many different professionals engaged in a strong multidisciplinary, academic, and humane team (from undergraduate students to Ph.D. professors). The shared vision of all the participants reported herein is expected to contribute to production of relevant scientific knowledge about mental health in a university context. The results will be published in the form of scientific articles exploring regional and nationwide realities from a multicenter and multilevel perspective.

Local and national seminars are planned, including a final report for undergraduate students and the university management. The SABES-Grad project helps promote mental health support and prevention through interventions at the selected institutions. Future prospects include repetition of the study to enable temporal assessment and epidemiological follow-up on mental health/suffering levels. Furthermore, the study is planned to expand to include other universities, creating a national survey representative of Brazilian universities.
