# Supplementary material for: A Study on the Health and Wellness of Undergraduate Students (SABES-Grad): methodological aspects of a nationwide multicenter and multilevel study overlapping with the Covid-19 pandemic
Source: Trends Psychiatry Psychother. 2023 Mar 7;45:e20210367. doi: 10.47626/2237-6089-2021-0367 (PMC10164404; doi:10.47626/2237-6089-2021-0367)
Supplement: Supplementary file 1 [file 2238-0019-trends-45-e20210367-suppl.pdf]

http://doi.org/10.47626/2237-6089-2021-0367

Table S1 - Outcome's description, instruments and variables operationalization. The *SABES-Grad* project. Brazil, 2021.

Outcome	Instrument	Operationalization
Generalized anxiety symptomatology	General Anxiety Disorders-7[16]	Score >15: Presence of symptoms of generalized anxiety disorder.
Depressive symptomatology	Patient Health Questionnaire-9[17]	Presence of clinically relevant depressive symptomatology according to the algorithm that uses internationally accepted diagnostic criteria[18]
Suicide risk	Mini Neuropsychiatric Interview[19] – adapted for self-administered use	Score = 0: No risk<br>Score 1-5: Low risk<br>Score 6-9: Moderate risk<br>Score ≥10: High risk
Use of alcohol, tobacco and illicit drugs	World Health Organization Methodology for Student Drug-Use Surveys[15, 20]	Lifetime use (experimentation), last-year use (past 12 months), and last-month use (past 30 days)
Stress	Perceived Stress Scale-14[21]	Total score (continuous variable): The higher the score, the higher the level of perceived stress.
Social anxiety (only in 2019 data collection)	Mini Social Phobia Inventory[22]	Score ≥ 6: Presence of symptomatology of social anxiety disorder.
Quality of life (only in 2019 data collection)	World Health Organization Quality of Life – Short version[23]	Total and domain (physical health, psychological, social relationships and environment scores: The higher the score, the higher the level of quality of life.

Reference numbering follows the same order as in the main text.

Table S2 - Individual and contextual exposures description, questions and instruments utilized and operationalization of the variables. The *SABES-Grad* project. Brazil, 2021.

**Social, economic, and behavioral characteristics**	
Biological sex	Male, female.
Gender identity	Male, female, non-binary, other.
Age	Years.
Weight	Self-reported (kilograms).
Height	Self-reported (centimeters).
Relationship status	Single, dating, has a partner or lives together, married, divorced, widowed.
Skin color	White, black, brown, yellow, other.
Religion	Presence of religion (yes, no), importance of religion (from none to maximum), frequency of attendance to religious ceremonies (from never to daily).
Income	Individual income (reais), family income (reais) and number of people that depends on the family income. Per capita family income was calculated by dividing total family income by the number of dependents.
Parents schooling level	Question about parents (mother and father) schooling level. Answers ranged from “did not attended to school” to “P.hD.”, including the possibility to respond “don’t know”, in the event of not knowing this information or not having contact with the family member.
Socioeconomic position and childhood adverse experience (only in 2019 data collection)	Questions from the Pró-Saúde study about place of residence, whether the parents were alive, who they lived with, economic situation, food insecurity, and experience of violence in the domestic context in childhood (when the person was 12 years old)[25].
Physical activity and sedentary behavior	Measured through the International Physical Activity Questionnaire (IPAQ) in the 2019 data collection[24]. Measured through two questions in the 2020/2021 data collection: “Considering the last 7 days, how many days did you do physical activity?”, and “On the days you did physical activity, how long did it last on average (in minutes)?”
Sleep quality	Measured through the Mini Sleep Questionnaire, translated and validated to use with Brazilian population[31]. The instrument asks about the frequency of sleep-related difficulties. Participants can be categorized into those with good sleep quality (score 10-24), or those with mild (score 25-27), moderate (score 28-30) or severe sleep difficulties (score ≥31)[31].

**Demographic, household and living situation, and current support**	
Academic migration	City/state prior to university entry versus city/state of current residence during undergraduate course.
Living situation	Living alone, living with family members, living with friends or peers.
Household type	Apartment, house, student household, boarding house, other.
Household status	Property owned, rented, leased or occupied.
Fear of violence in the neighborhood	Five-point Likert-type answers ranging from “none” to “very much fear”
Adequate access to essential services	Water, electricity, sewage and garbage collection.
Perception of access to and use of medical and psychological professionals' services	Questions were asked about the perception of access to physicians and psychologists. The possible responses were the five-point Likert-type answers, ranging from “very easy” to “very difficult”. As for use, a question was asked about the last time the participant used this service (ranging from “less than a week ago” to “never”).
Social support	Through the Social Support Scale, translated and validated for Brazil[29]. Individuals can be categorized as with low, medium or high social support[30].

**Academic characteristics**	
Undergraduate course	Questions regarding undergraduate course currently taken, if this is the first undergraduate course of the individual, and if the participant had initiated and/or concluded another undergraduate-level course.
Shift	Morning, afternoon, night
Year of entry	Question about what year the individual entered the university (to take the current course).
Course duration	In years and/or semesters
Current year/semester	Question about which year/semester the participant was currently coursing.
Number of failures	Question about the number of failures in the last semester. Possible answers were “none”, “one”, “two”, “three or more” or “I haven't been evaluated yet” (for those who were in the first semester).
Desired undergraduate course	Question to assess whether the current course was the one the participant wanted when entering the university.
Satisfaction with current undergraduate course	Question about the level of satisfaction with the current course, with a Likert-type response ranging from “not at all satisfied” to “totally satisfied”.

**Sexual behavior and sexually transmitted infections (STI)**	
Sexual orientation	Heterosexual, homosexual, bisexual, asexual, pansexual or otherwise.
Lifetime sexual intercourse	Question about lifetime experience (or absence) of sexual intercourse.
Age at first sexual intercourse	Years.
Age (even if approximate) of partner at first sexual intercourse	Years.
Consent at first sexual intercourse	Yes, no.
Forced sexual intercourse experience	Yes, no (if yes, how many times).
Presence of fixed and non-fixed sexual partners in the last 12 months	Yes, no (if yes, how many).
Frequency of condom use with fixed and non-fixed sexual partners in the last 12 months	Five-point Likert-type response, ranging from “never”, “less than half the time”, “half the time”, “more than half the time”, and “always”.
Condom use at last sexual intercourse	Yes, no, don’t remember.
Lifetime and last-year (past 12 months) diagnosis of Sexually Transmitted Infections (STI)	Questions about lifetime and last-year (past 12 months) diagnosis of the following STI: chancroid, Human papillomavirus (HPV), Gonorrhea, Chlamydia, Syphilis, Genital Herpes and Human Immunodeficiency Virus (HIV).
**Current adverse experiences**	
Discrimination	Through the Everyday Discrimination Scale, translated and validated for Brazil[27]. Questions were asked about lifetime experience of discrimination, both within the university and in other settings (public places, work, housing or police). For each question, the source by which the participant believes they have been discriminated against were asked: skin color, gender, religion, disease, sexual orientation, economic status, political activity, age or physical appearance. Finally, individuals were asked about the last time the discrimination happened (less than a month ago, between one and 12 months ago, or more than 12 months ago).
Food insecurity	Through a reduced version of the Brazilian Food Insecurity Scale[28]. All participants with one point or more in this questionnaire were considered to be food insecure.
**Covid-19 pandemic (only in 2020/2021 data collection)**	
Impacts of the pandemic on occupation/work	Question about how the Covid-19 pandemic affected occupation/work, with the following answer alternatives: "I didn't work before and I

	continued without working", "I continued working normally", "I continued working, but at home (Home Office)" , "I started working during the pandemic", "I lost my job or stopped working", or "other".
Impacts of the pandemic on family income	Question about how the Covid-19 pandemic affected the family income, with the following answer alternatives: "It increased a lot", "It increased a little", "It was kept the same", "It decreased a little", "It decreased a lot", or "We were without income".
Compliance with social distancing recommendations	Question on how much the participant thought he was complying with the recommendations on social distancing, with the following answer alternatives: "Very little", "little", "more or less", "fairly" or "practically isolated from everyone".
Activities routine during the pandemic	Question about how the participant's routine has been during the Covid-19 pandemic, with the following answer alternatives: "I stay at home all the time", "I only go out for essential activities, such as buying food and medication", "I go out sometimes for non-essential activities", "I go out every or almost every day for non-essential activities", or "I go out every or almost every day for work or other regular activity".
Inflow and outflow of people in the house	Question about how the inflow and outflow of people in the participant's home has been during the Covid-19 pandemic, with the following answer alternatives: "Only I have entered my house", "Only family members who live together", "Some close relatives visit once or twice a week", "Some close relatives visit almost every day", "Friends, relatives or others visit once or twice a week", or "Friends, relatives or others visit almost every day".
Number of days left home in the last 15 days	Number of days (zero to 15).
Access to information	Questions about how many days the participant accessed information about the pandemic in the last week; approximate number of times the individual sought information about Covid-19 on the days he/she accessed information; and the average time, in minutes, that the individual spent when accessing information about Covid-19. Using this three variables, it was possible to calculate the total time of access to information per week (number of days x number of times x number of minutes).
Alterations in sleep due to the Covid-19 pandemic	Two questions were asked: one comparing the changes in sleep quality ("It got a lot worse", "It got a little worse", "Still the same quality", "It improved a little" and "It improved a lot"), and another in relation to the sleep duration ("It didn't change" , "I started sleeping more" or "I started sleeping less") comparing the pre- and ongoing pandemic period.
Fear of Covid-19	Through the Fear of Covid-19 Scale[32], translated and validated for Brazil[33, 34]. It is an instrument with seven statements about aspects related to fear and anxiety reactions to the disease, with Likert-type answers of 5 points (1 to 5). The total score varies between 7 and 35

	points, with participant being classified as with low fear (7 to 19), moderate fear (20 to 26), and very much fear (27 or more)[33].
Test, result and outcomes related to Covid-19 infection	Question about testing for Covid-19 infection. If affirmative, it was followed by a question about the test result (positive or negative). For positive cases, questions were also asked about related outcomes (staying at home, care by teleservices, visit by a health professional, hospitalization, admission to the Intensive Care Unit (ICU)).
Presence of risk factors for Covid-19	Questions about the presence of the following risk factors: age (65 years or older), hypertension, diabetes, high cholesterol or triglycerides, heart disease, history of stroke, cancer, respiratory problems, and/or obesity.
Infection and/or death of loved ones by Covid-19	Yes, no (if yes, possibilities were mother, father, brother, grandparents, children, other relative, friend and/or partner).

Reference numbering follows the same order as in the main text.
